# Identification of *Mycobacterium tuberculosis *clinical isolates in Bangladesh by a species distinguishable multiplex PCR

**DOI:** 10.1186/1471-2334-10-118

**Published:** 2010-05-15

**Authors:** Chie Nakajima, Zeaur Rahim, Yukari Fukushima, Isamu Sugawara, Adri GM van der Zanden, Aki Tamaru, Yasuhiko Suzuki

**Affiliations:** 1 Department of Global epidemiology, Hokkaido University Research Center for Zoonosis Control, Kita20-Nishi10, Kita-ku, Sapporo 001-0020, Japan; 2 Tuberculosis laboratory, International Centre for Diarrhoeal Disease Research, Bangladesh (ICDDR, B), GPO Box 128, Dhaka 1000, Bangladesh; 3 Research Institute of Tuberculosis, Japan Anti-Tuberculosis Association, 3-1-24 Matsuyama, Kiyose, Tokyo, Japan; 4 Laboratory for Medical Microbiology and Public Health, P.O.Box 377, Burg. Edo Bergsmalaanl, 7512 AD Enschede, The Netherlands; 5 Osaka Prefectural Institute of Public Health, 1-3-69, Nakamichi, Higashinari-ku, Osaka 537-0025, Japan

## Abstract

**Background:**

Species identification of isolates belonging to the *Mycobacterium tuberculosis *complex (MTC) seems to be important for the appropriate treatment of patients, since *M. bovis *is naturally resistant to a first line anti-tuberculosis (TB) drug, pyrazinamide, while most of the other MTC members are susceptible to this antimicrobial agent. A simple and low-cost differentiation method was needed in higher TB burden countries, such as Bangladesh, where the prevalence of *M. bovis *among people or cattle has not been investigated.

**Methods:**

Genetic regions *cfp32*, RD9 and RD12 were chosen as targets for a species distinguishable multiplex PCR and the system was evaluated with twenty reference strains of mycobacterial species including non-tubercular mycobacteria (NTM). A total of 350 clinical MTC isolates obtained in Bangladesh were then analyzed with this multiplex PCR.

**Results:**

All of the MTC reference strains gave expected banding patterns and no non-specific amplifications were observed in the NTM strains. Out of 350 clinical isolates examined by this method, 347 (99.1%) were positive for all of the *cfp32*, RD9 and RD12 and determined as *M. tuberculosis*. Two isolates lacked *cfp32 *PCR product and one lacked RD12, however, those three samples were further examined and identified as *M. tuberculosis *by the sequence analyses of *hsp65 *and *gyrB*.

**Conclusions:**

The MTC-discrimination multiplex PCR (MTCD-MPCR) developed in this study showed high specificity and was thought to be very useful as a routine test because of its simplicity. In the current survey, all the 350 MTC isolates obtained from Bangladesh TB patients were determined as *M. tuberculosis *and no other MTC were detected. This result suggested the general TB treatment regimen including pyrazinamide to be the first choice in Bangladesh.

## Background

*Mycobacterium tuberculosis *complex (MTC), including *M. tuberculosis*, *M. bovis*, *M. africanum, M. microti, M. pinnipedii, M. caprae*, "*M. canettii" *and other closely related strains, is a group of causative agents for human and animal tuberculosis (TB) [1,2]. Although the mycobacterial species in MTC are highly similar to each other in DNA level, MTC members differ widely in terms of host tropism, phenotype and pathogenicity [1,3-5]. No further differentiation is usually performed with isolates determined as MTC, however, it seems to be important in some cases for the appropriate management of patients or for an epidemiological purpose. Especially, in the case of *M. bovis *infection, to identify the species in the early stage of diagnosis is essential to avoid inappropriate treatment, since *M. bovis *is naturally resistant to a major anti-TB drug, pyrazinamide [3,4,6], and the standard regimen including this drug has to be altered.

Several rapid identification methods using nucleic acid amplification techniques have been developed and used for the diagnosis of TB [7-9], however, they do not differentiate *M. tuberculosis *from other members of MTC. Recent comparative genomic analyses have provided valuable information on the region of difference (RD) in the chromosome of MTC to indicate that specific identification of MTC can be achieved by the detection of these regions [1-3,10]. PCR-based methods targeting RDs can be easily performed in local clinical laboratories with low expense [3].

Bangladesh is one of the highest TB burden countries, where the estimated number of TB incidence in 2007 was 353,000 to be ranked sixth in the world in the WHO report [11]. In this country, a large number of cattle estimated 23 million heads are reared in households especially in rural areas [12]. Some surveys about drug resistant *M. tuberculosis *[13,14] or *M. tuberculosis *epidemiology [15] have been performed, however, no survey about *M. bovis *prevalence among humans as well as cattle has been reported though people are living in a close relationship with cattle [12,16].

In this study, we developed a simple multiplex-PCR system, named MTC-discrimination multiplex PCR (MTCD-MPCR), to distinguish *M. tuberculosis *from other MTC species using RDs, and applied it for clinical isolates derived from TB patients in Bangladesh.

## Methods

### Bacterial strains and sample collection

For the evaluation of the method, following twenty reference strains, four MTC strains and sixteen nontuberculous mycobacteria (NTM) strains, were obtained from the Research Institute of Tuberculosis, Japan Anti-Tuberculosis Association (Tokyo, Japan) and used: *M. tuberculosis *H37Rv, *M. africanum *KK13-02, *M. microti *ATCC19422, *M. bovis *BCG Tokyo, *M. avium *JATA51-01, *M. intracellulare *JATA52-01, *M. kansasii *KK21-01, *M. xenopi *KK42-02, *M. fortuitum *JATA61-01, *M. lentiflavum *JATA9N-01, *M. simiae *KK23-01, *M. gordonae *JATA33-01, *M. marinum *JATA22-01, *M. asiaticum *KK24-01, *M. scrofulaceum *JATA31-01, *M. szulgai *JATA32-01, *M. nonchromogenicum *JATA45-01, *M. malmoense *JATA47-01, *M. chelonae *JATA62-01 and *M. abscessus *JATA63-01.

Clinical samples were collected in hospitals in Dhaka, located in an urban area, and Matlab and Sylhet, located in rural areas. A total of 350 isolates, 300 from Dhaka, 41 from Matlab and 9 from Sylhet, were examined (Additional file 1). Among them, 327 isolates were derived from sputa, 22 were from lymph nodes and remaining 1 was from a surgical injury.

### Cultivation and biochemical characterization of isolates and DNA extraction

Sputa and other samples were collected from TB suspected patients and decontaminated following the Petropff's method [17]. After a centrifugation at 500 rpm at 4°C, the supernatant was discarded and one loop-full (5 mm diameter of the loop) decontaminated pellet was inoculated onto 2 Lowenstein-Jensen (L-J) slants each. Inoculated L-J slants were incubated at 37°C. Each L-J slant was examined once a week for contamination as well as for growth until 8 weeks. Typical mycobacterium-like colonies were tested for sensitivity to p-nitrobenzoic (PNB) acid. PNB sensitive strains were considered to be *M. tuberculosis *complex. DNA was extracted from those colonies by heating at 95°C for 5 min followed by chloroform extraction and ethanol precipitation [18].

### MTC-discrimination multiplex PCR (MTCD-MPCR)

Primer pairs for *cfp32 *(Rv0577), RD9 (Rv2073c) and RD12 (Rv3120) designed by Huard *et al *[3] were slightly modified and used as a primer mixture for three simultaneous PCRs in one tube (Table 1). The reaction mixture contained 1 mM dNTPs (0.25 mM each), 0.5 M betaine, 750 nM each of *cfp32 *primers (Rv0577F and Rv0577R), 250 nM each of RD primers (Rv2073cF, Rv2073cR, Rv3120F and Rv3120R (390-369)), 1 μL of DNA sample and 1 U of GoTaq DNA Polymerase (Promega Corp., WI, U.S.A.) in 20 μL of Green GoTaq Reaction Buffer. PCR reaction was initiated by denaturation for 1 min at 96°C, followed by 35 cycles of 10 s at 96°C, 20 s at 60°C and 1 min at 72°C with final extension for 5 min at 72°C in a thermalcycler (iCycler, Bio Rad Laboratories Inc., CA, U.S.A.). Reaction mixtures with *M. tuberculosis *DNA and without template DNA were also run simultaneously with samples every time as a positive control and a negative control to evaluate the MTCD-MPCR system. The products were electrophoresed in 2.0% agarose gel in TAE buffer, and stained with ethidium bromide.

**Table 1:** Used primers for the MTCD-MPCR and additional PCRs and sequencings.

Target locus	Primer name	Primer sequence	Location^*a*^	Size (bp)	Ref. No.
**MTCD-MPCR**					
*cfp32*	Rv0577F	5' ATGCCCAAGAGAAGCGAATACAGGCAA	671166-192	786	[3]
	Rv0577R	5' CTATTGCTGCGGTGCGGGCTTCAA	671951-928		
RD9	Rv2073cF	5' TCGCCGCTGCCAGATGAGTC	2330579-598	600	[3]
	Rv2073cR	5' TTTGGGAGCCGCCGGTGGTGATGA	2331173-150		
RD12	Rv3120F	5' GTCGGCGATAGACCATGAGTCCGTCTCCAT	3485558-587	404	[3]
	Rv3120R (390-369)	5' GCGAAAAGTGGGCGGATGCCAG	3485961-940		
**Additional PCRs or sequencings**					
*cfp32*	3'cfp32F	5' CGAATCATTGGCACGTCTACTTTG	671770-793	372	[2]
	3'cfp32R	5' GTGGCACCGGCGGCACCGCACACCT	672141-117		
RD12	Rv3120-F (90-110)	5' GGTATTTGCGCCCATATCCTG	3485661-681	411	this study
	Rv3120-R (500-481)	5' CCTGGCTTCAAGCACCATTC	3486071-052		
*rpoB*	*rpoB*-F^*b*^	5' CAGGACGTGGAGGCGATCAC	761007-026	250	[18]
	*rpoB*-R	5' CAGGGGTTTCGATCGGGCAC	761256-237		
*rpoB *^*c*^	*rpoB*-S-F^*b*^	5' GCGTACGGTCGGCGAGCTGATCC	760922-944	418	this study
	*rpoB*-S-R	5' GCGGTACGGCGTTTCGATGAACC	761339-317		
*rrs*	Bact-*rrs*-F^*b*^	5' AGAGTTTGATCCTGGCTCAG	1471856-875	1496	this study
	Bact-*rrs*-R	5' TACGGCTACCTTGTTACGAC	1473351-332		
*rrs*^*c*^	*rrs*-S-F^*b*^	5' ATACCTTTGGCTCCCTTTTCC	1471809-829	1607	this study
	*rrs*-S-R	5' CCCACCAGTTGGGGCGTTTTC	1473415-395		
*hsp65*	*hsp65*F^*b*^	5' ACCAACGATGGTGTGTCCAT	528752-771	441	[2]
	*hsp65*R	5' CTTGTCGAACCGCATACCCT	529192-173		
*gyrB*	*gyrB*F^*b*^	5' ACATCAACCGCACCAAGAACGC	6027-048	483	this study
	*gyrB*R	5' GTGCCTTACGTGCCGCGATACG	6509-488		

^*a *^Location on the *M. tuberculosis *H37Rv genome (accession no. NC_000962.2).

^*b *^These primers were used for PCR and sequencing.

^*c *^Those primer pairs were used for the samples that showed non-MTC gene sequences.

The sensitivity of the method was determined using serially diluted purified genomic-DNA solutions, ten-fold dilution from 5 ng/μL to 50 fg/μL, extracted from *M. tuberculosis *H37Rv and *M. bovis *BCG Tokyo. For the specificity study, the concentration of the DNA solution from each reference strain was adjusted to 5 ng/μL and used.

A detection study from sputum samples was performed with *M. tuberculosis *H37Rv spiked sputa. Serially diluted bacteria were spiked into healthy volunteer's sputum samples to final concentrations ranging from 1.5 × 10/mL to 1.5 × 10^6^/mL. The sputum was processed by a conventional method and DNA was extracted by bead-beating. Briefly, twice the volume of N-acetyl-L-cysteine and NaOH (NALC-NaOH) was added to the sputum, mixed well and incubated for 15 minutes. The sample was diluted to five times its original volume with PBS and centrifuged for 20 min at 3000 rpm. The sediment was dissolved in 0.75 mL of Tris-EDTA buffer, added 0.5 g of glass beads (0.15 - 0.25 mm, Fuji Chemical Industry Co., Ltd., Japan) and 0.5 mL of chloroform and then shaken with a bead-beater (FastPrep FP100A, MP Biomedicals, U.S.A.) for 5 min at 5500 rpm. Tubes were centrifuged at 5000 rpm for 5 min and 0.5 mL of PCI (phenol-chloroform-isoamyl alcohol, 25:24:1) was added to the supernatant, mixed and centrifuged. DNA was extracted from the PCI treated sample by isopropyl alcohol precipitation and the precipitant was dissolved in 10 μL of Tris-EDTA buffer. Final solutions were subjected to the MTCD-MPCR.

### Other PCRs and Sequence analyses

Additional PCRs and sequencings were performed with primers listed in Table 1. Reaction solution components in those PCRs were as follows: 1 mM dNTPs (0.25 mM each), 0.5 M betaine, 500 nM each of forward and reverse primers, 1 μL of DNA sample and 1 U of GoTaq DNA Polymerase (Promega Corp.) in 20 μL of Green GoTaq Reaction Buffer. PCR reaction other than *rrs *was initiated by denaturation for 1 min at 96°C, followed by 30 cycles of 10 s at 96°C, 10 s at 55°C and 30 sec at 72°C with final extension for 5 min at 72°C in a thermalcycler (iCycler, Bio Rad Laboratories Inc.). In *rrs *PCR, the period of extension at 72°C in the cycle was 90 sec, whereas other conditions were same with other PCR procedure. The products were electrophoresed in 1.5% agarose gel and stained with ethidium bromide. Sequencing of PCR product was performed according to manufacturer's protocol with ABI PRISM 3130*xl *Genetic Analyzer (Life Technologies Corp., CA, U.S.A.) using BigDye Terminator v3.1 Cycle Sequencing Kit (Life Technologies Corp.). *rpoB *and *rrs *sequences read by each forward primer (Table 1. *b*) were compared with the sequence of *M. tuberculosis *H37Rv and the samples met following criteria were identified as MTC: *rpoB*, more than 98% match in minimum 150-base length; *rrs*, 100% match in minimum 300 bases. About the samples determined as non-MTC by these criteria, another PCR and sequencing were done with more MTC specific primers (Table 1. *c*) with the same criteria. The samples identified as MTC with those specific primers were determined as mixed-culture samples. Species identifications by *hsp65 *or *gyrB *sequences were done according to previous publications [2,19,20].

### Ethical Clearance

The original research project was approved by the Research Review Committee and Ethical Review Committee of the International Centre for Diaddroeal Disease Research, Bangladesh (ICDDR, B). Signed informed consent was obtained from each patient and volunteer recruited for the study.

## Results and discussion

### MTCD-MPCR

**Figure 1:**
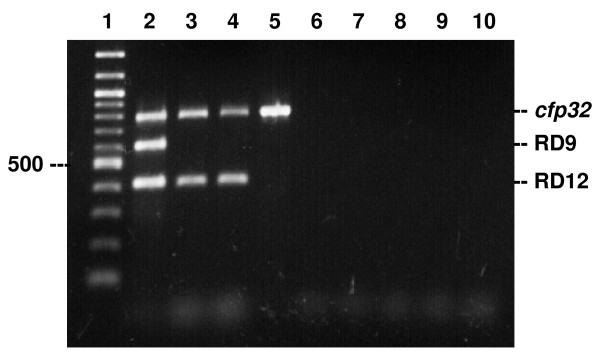
**Typical MTCD-MPCR banding patterns of mycobacterial reference strains**. PCR products by the MTCD-MPCR were analyzed on 2.0% agarose gel electrophoresis followed by ethidium bromide stain. Lanes: 1, 100-bp ladder; 2, *M. tuberculosis*; 3, *M. africanum*; 4, *M. microti*; 5, *M. bovis*; 6, *M. kansasii*; 7, *M. avium*; 8, *M. intracellulare*; 9, *M. fortuitum*; 10, negative control

Three genetic regions were selected as the targets for the multiplex PCR: *cfp32*, RD9 and RD12. *cfp32 *is an MTC-restricted gene and used to confirm isolates to belong to MTC [3,21]. RD9 is the region that can be found in only *M. tuberculosis *and "*M. canettii"*, and RD12 is found in all MTC members except *M. bovis*, *M. caprae *and "*M. canettii" *[2,3]. By a trial with several patterns of primer concentrations, the best combination was determined to be 750 nM each of *cfp32 *primers and 250 nM each of RD9 and RD12 primers in the multiplex PCR. With this PCR, an isolate possessing all the three regions can be identified as *M. tuberculosis *whereas a strain showing only one amplified band, *cfp32*, will be classified as *M. bovis *or *M. caprae *(Figure 1, Table 2). Other banding patterns are interpreted as described in Table 2. "*M. canettii" *is another MTC that clinical isolates reported so far were naturally pyrazinamide resistant [6,19]. Thus, this PCR system was thought to be useful for the discrimination of MTC species, especially for the screening of naturally pyrazinamide-resistant species with only one PCR reaction per sample.

**Table 2:** Results of the MTCD-MPCR with Bangladesh clinical isolates.

Species Interpretation	Banding pattern ^*a*^			Number of isolates	%
	*cfp32*	RD9	RD12		
*M. tuberculosis*	+	+	+	347	99.1
*M. bovis/M. caprae*	+	-	-	0	0
*"M. canettii"*	+	+	-	1 ^*c*^	0.3
Other MTC ^*b*^	+	-	+	0	0
Non MTC	-	-	-	0	0
Irregular	-	+	+	2 ^*d*^	0.6

Total				350	100

^*a *^Banding patterns are shown as amplification results of the MTCD-MPCR in order of *cfp32*, RD9 and RD12: +, Amplification positive; -, negative.

^*b *^Other MTC includes following species: *M. africanum, M. microti, M. pinnipedii*, dassie bacillus and oryx bacillus. The latter two are minor strains belonging to MTC [2].

^*c *^Confirmed to be *M. tuberculosis by hsp65 *and *gyrB *sequencing.

^*d *^Confirmed to be *M. tuberculosis by gyrB *sequencing.

The sensitivities of the method were determined as 500 fg genomic DNA for *M. tuberculosis *(H37Rv) and 50 fg for *M. bovis *(BCG Tokyo), which were assumed to be equivalent to 100 bacilli and 10 bacilli, respectively (data not shown). The specificity of the MTCD-MPCR was confirmed with DNA templates extracted from mycobacterial reference strains. Typical gel electrophoresis results of the MTC and NTM strains are shown in Figure 1. All of the PCR products from the MTC strains gave expected banding patterns in correct sizes (Table 1). On the contrary, no bands were obtained from the NTM samples. These results demonstrated the specificity and applicability of the MTCD-MPCR for the differentiation of *M. tuberculosis *in clinical isolates.

In the study using bacterium-spiked sputa, the detection limit of *M. tuberculosis *was 1.5 × 10^4 ^cells in 1 mL of sputum (data not shown). This bacterial concentration is similar or a little higher than the detection limit of the bacteria by the Zeal-Nelsen staining. This means species discrimination by the MTCD-MPCR is applicable for clinical samples if the sputum is diagnosed as smear positive. The discrepancy of the detection limit between purified DNA and bacterium-spiked sputum seems to depend on the extraction or purification procedure, which is to be improved.

### Discrimination of MTC in Bangladesh clinical isolates

A total of 350 clinical isolates obtained in Bangladesh were analyzed to see the prevalence of MTC species other than *M. tuberculosis*. All samples were subjected to *rpoB *or *rrs *sequencing to confirm as MTC [22]. By this sequence analysis, 18 samples were revealed as mixtures of MTC and other bacteria, mainly mycobacterial species. Those mixed samples were also subjected to the MTCD-MPCR study.

Out of the 350 isolates, 347 (99.1%) showed the typical banding pattern of *M. tuberculosis *in the electrophoresis by the MTCD-MPCR (Table 2). Among three remaining isolates, one (0.3%, isolate ATP138) lacked RD12 band showing "*M. canettii*" pattern and two (0.6%, isolate M2000 and S2247) did not have *cfp32 *band, presented as irregular in Table 2. Those samples were further analyzed to detect the target region, *cfp32 *or RD12, with other sets of primers (Table 1) [2]. With this trial, sample S2247 gave an expected sized *cfp32 *band, suggesting a partial deletion or a mutation in the primer-binding site. Other two samples, ATP138 and M2000, still failed to amplify the expected DNA fragment indicating that larger deletion event had occurred in the target genomic area. ATP138, the "*M. canettii*" pattern sample, was subjected to *hsp65 *sequencing and confirmed not to be "*M. canettii*" [2,19]. These three isolates were further confirmed to be *M. tuberculosis *by the sequencing of *gyrB *[20]. No isolates out of the 350 lacked RD9 band, concordant with the observations by former researchers [2,10,23], possibly indicating the high stability of this region in *M. tuberculosis*. The lack of *cfp32 *in an MTC isolate has been reported by Huard *et al *[2] with a similar incidence (1/125, 0.8%) to the current study. Some of the mixed-culture samples showed correct but faint banding patterns, suggesting an inhibitory effect of contaminated DNA. The result indicated that despite its decreased sensitivity, the MTCD-MPCR was able to detect MTC from mixed-culture samples, which sometimes are observed in primary cultures [8].

All of the 350 MTC isolates obtained from clinical specimens in Bangladesh were *M. tuberculosis *and no other MTC species were detected. This information is very helpful for the management of patients to determine treatment regimens. Patients suffered from MTC in surveyed area in Bangladesh can possibly be subjected to the standard regimen including pyrazinamide. Although *M. bovis *was not detected from human in current study, there are no precise data about *M. bovis *prevalence among cattle in this country, continuous surveys seem to be needed especially in rural areas where people and cattle inhabit more closely [12]. Since pyrazinamide is suggested to have more adverse side effects than other first-line anti-TB drugs [24], useless administration to patients should be avoided.

The MTCD-MPCR developed in this study is considered to be very useful for the differentiation of MTC because of its simplicity and specificity. A large number of samples can be analyzed by this method in a short period of time. Some other MTC discrimination methods using RDs have been published and showed higher differentiation capability that could distinguish almost all members of MTC. However, the procedures were more time consuming (e.g., multiple PCR reactions were needed) [2,3], result interpretations seemed to be complicated (e.g., sizes of amplified bands should be estimated) [10,23,25] or an expensive equipment should be needed [26]. The necessity of detailed MTC discrimination seems to be low since the majority of human tuberculosis causing agents are *M. tuberculosis*, and in some global areas, *M. bovis *partially contributes to the prevalence [4,23]. The vaccine strain bacillus Calmette-Guerin (BCG), an attenuated *M. bovis*, can be a cause of disseminated mycobacterial infection in immunocompromised individuals, however, the patients can be treated by the same regimen as *M. bovis *without pyrazinamide [27], and if necessary, an additional PCR for the detection of RD1 can distinguish BCG from clinical *M. bovis *strains [3]. Possibility of the detection of other MTC species is almost negligible in routine laboratory diagnoses, except for the cases in some African countries where *M. africanum *can be found in a higher ratio [2]. Samples exhibiting rare or irregular banding patterns by the MTCD-MPCR can be examined afterward using other precise methods, e.g., targeting other RDs, gene sequencing or spoligotyping [2,19,20,28]. Thus, the MTCD-MPCR, a simple MTC discrimination method developed and evaluated in this study seemed to be very useful as a screening tool for clinical isolates to distinguish *M. tuberculosis *from *M. bovis *for the prompt decision of treatment regimen.

## Conclusions

In the current study, the MTC-discrimination multiplex PCR (MTCD-MPCR) was developed and applied for a study to see the prevalence of MTC species other than *M. tuberculosis *in clinical isolates in Bangladesh. The method showed high specificity and sensitivity, as 99.1% (347/350) of clinical *M. tuberculosis *isolates were identified by a typical banding pattern. It seemed to be very useful as a routine test method because of its simplicity. All the 350 MTC isolates derived from Bangladesh patients were *M. tuberculosis *and no other MTC was detected. The result suggested that a standard TB treatment regimen including pyrazinamide can be applied to the patients as the first choice in surveyed areas in Bangladesh.

## Competing interests

The authors declare that they have no competing interests.

## Authors' contributions

CN, ZR and YS were responsible for planning the study, analyzing the results and drafting the manuscript. YF carried out the molecular genetic studies. AT performed the detection study from sputum samples. ZR, IS, AGMZ and YS collected the study material and coordinated the study. All authors read and approved the manuscript.

## Pre-publication history

The pre-publication history for this paper can be accessed here:

http://www.biomedcentral.com/1471-2334/10/118/prepub

## Supplementary Material

Additional file 1 **Used isolates in current study**. Background information of used isolates including sampling site and drug-susceptibility test results. Click here for file
